# Liraglutide and Metformin alone or combined therapy for type 2 diabetes patients complicated with coronary artery disease

**DOI:** 10.1186/s12944-017-0609-0

**Published:** 2017-12-02

**Authors:** Ying Liu, Xia Jiang, Xin Chen

**Affiliations:** 1 Department of Endocrinology, Tianjin First Center Hospital, 24 Fukang Road, Nankai District, Tianjin, 300192 China; 2 Department of Cardiovascular Medicine, Tianjin First Center Hospital, Tianjin, 300192 China

**Keywords:** Liraglutide, Metformin, GLP-1, Type 2 diabetes mellitus (T2DM), Coronary artery disease (CAD)

## Abstract

**Background:**

This study is to compare the effects of Liraglutide and Metformin alone or combined treatment on the cardiac function in T2DM patients complicated with CAD.

**Methods:**

120 T2DM patients were included at Endocrinology Department of Tianjin First Center Hospital (Tianjin, China) from April 2012 to September 2013. The study contained two sections. *Section 1:* 30 patients in group 1 was treated with Liraglutide (Novo Nordisk) (1.2 mg/d), and 30 patients in group 2 with Metformin (Shiguibao) (1500 mg/d) for 24 weeks. *Section 2:* 30 patients in group1 was treated with Liraglutide (1.8 mg/d) and 30 in group 2 with Liraglutide (1.2 mg/d) plus Metformin (1500 mg/d) for 24 weeks. Fasting blood glucose (FBG), postprandial glucose (PPG), glycated hemoglobin (HbA1c), body mass index (BMI), blood pressure (BP), triglyceride (TG), total cholesterol (TC), low-density lipoprotein cholesterol (LDL-C), C reactive protein (CRP), left ventricular end-diastolic diameter (LVEDD), ejection fraction (EF) and the ratio of early (E) to late (A) ventricular filling velocities (E/A ratio) were measured before and after the 24-week treatment.

**Results:**

After 24-week treatment, when blood glucose level was controlled in 4 groups, Liraglutide alone treatment showed better improvements than on all measuring except TG in Section 1, however, combined treatment of Liraglutide and Metformin showed better improvements on all measuring except BMI, TG and BP in Section 2.

**Conclusions:**

With similar glycemic control, the Liraglutide (1.2 mg/d) monotherapy showed the better effects than either Metformin alone, or combination of Liraglutide and Metformin on lipid metabolism and cardiovascular function.

**Trial registration:**

This trial was registered at Chinese Clinical Trial Registry (chictr.org.cn) # ChiCTR-IPR-16008578.

## Background

Type 2 diabetes mellitus (T2DM) is commonly complicated with coronary artery disease (CAD), hypertension, stroke and heart failure [1]. As a result of small vessel disease and metabolic disorders, diabetes is associated with some forms of cardiomyopathy and congestive heart failure [2]. On the other hand, CAD accounts for about 80% of deaths in T2DM patients. Besides the glucose level, other risk factors include hypertension, dyslipidaemia, obesity and insulin resistance [3]. Although glycemic control represents the classical goal of diabetes therapy, the pathogenesis of the cardiovascular complications extends beyond hyperglycemia. Thus, from an individualized perspective, apart from glycemic control, improvement of cardiovascular function should also be taken into account for T2DM patients complicated with CAD.

Glucagon-like peptide-1 (GLP-1) is an endogenous insulinotropic peptide produced by intestinal epithelial endocrine L-cells and is considered to potentiate the incretin effect [4]. GLP-1 receptor agonists (RAs) improve glucose homeostasis through multifaceted action. Liraglutide, an analogue of GLP-1, maintains glucose homeostasis through the regulation of insulin and glucagon secretion [5]. Liraglutide also regulates hypertension, left ventricular function, cardiac steatosis, oxidative stress, and apoptosis both in human and animal models [6–8]. Metformin is the first-line medication for the treatment of type 2 diabetes, and induces muscles to take up glucose from the blood. Metformin has also been reported to reduce the multiple CAD risk factors such as free fatty acid (FFA), triglyceride (TG) and remnant lipoprotein cholesterol (RLP-C) [9]. Animal studies also demonstrated a protective role of Metformin in myocardial dysfunction [10]. Moreover, it was reported that GLP-1 infusion improves left ventricular ejection fraction and functional status in patients with chronic heart failure [11].

Thus, we investigated the efficacy of alone or combined therapy of Liraglutide or Metformin on the cardiovascular function of T2DM patients complicated with CAD.

## Methods

### Study design

We conducted a pilot study with section 1 and 2. In section 1, 30 patients with T2DM were in Liraglutide monotherapy (1.2 mg/d) group and 30 patients with T2DM were in Metformin monotherapy (1500 mg/d) group. In section 2, 30 patients with T2DM were in Liraglutide monotherapy (1.8 mg/d) group and 30 patients with T2DM were in Liraglutide (1.2 mg/d) plus Metformin (1500 mg/d) group at the Department of Endocrinology of Tianjin First Center Hospital between April 2012 and September 2013. The study was approved by Ethics Committee of Tianjin First Center Hospital (No. 2014N0016LW), and was registered at Chinese Clinical Trial Registry (chictr.org.cn, No. ChiCTR-IPR-16008578). All patients provided written informed consent.

### Patients

The diagnostic criteria for diabetes were: oral glucose tolerance test, fasting blood glucose ≥7 mmol/L, 2-h postprandial glucose (PPG) ≥11.1 mmol/L and HbA1c ≥6.5% or 48 mmol/L. According to Clinical Practice Guidelines by European Society of Cardiology in 2006 (coronary artery stenosis on angiography), the diagnosis of CAD included one of the following: 1) having the typical angina symptom and excluding aortic valve lesion; 2) having history of old myocardial infarction; 3) having history of acute myocardial infarction; 4) coronary angiography revealed that > = 70% of coronary artery stenosis. Exclusion criteria were: 1) Type I diabetes; 2) history of tuberculosis, viral hemorrhagic fevers, avian influenza, viral hepatitis, atypical pneumonia, dysentery, meningococcal meningitis, or any other chronic or acute infectious diseases that affect carbohydrate metabolism within the past 6 months; 3) present illness that interferes with carbohydrate and/or lipids metabolism (such as hormones); 4) severe heart failure, cardiac hypertrophy, rheumatic heart disease, valve disorder, myocarditis or endocarditis, or any other significant cardiovascular events; 5) not suitable for coronary angiography; 6) women during pregnancy, lactation, or planning for pregnancy; 7) taking diet pills within the past 6 months; 8) chronic liver dysfunction (alanine aminotransferase/aspartate aminotransferase >2.5 ULN) and/or chronic renal dysfunction (serum creatinine >150 μmol/L); 9) severe electrolyte disorders; 10) previous episodes of acute orchronic pancreatitis; 11) history of tumor or any other severe diseases that are not suitable to be included as judged by the investigator.

### Treatment plans

Drugs used in the study included Liraglutid, 18 mg/3 ml/pen (pre-filled pen), (Novo Nordisk, Beijing, China) and Metformin (Glucophage) 0.5 mg/pill (Shanghai Shiguibao pharmaceutical Co. LTD, Shanghai, China).

All patients were instructed by the investigator for food intake, exercise, drug administration, fingertip blood glucose (FBG) measurement (Bayer Blood Glucose Monitoring System), and records of treatment for hypoglycemia and/or any adverse effects. All preexisting treatment for hypertension, hypercholesterolemia, anticoagulant and coronary dilation were maintained throughout the study. In addition, food intake was controlled and regular exercise was maintained. No drugs that potentially interfered with the outcomes were used. Ultrasound cardiography (UCG) and blood pressure were measured at the hospital.

Two-drug effects on cardiovascular diseases in T2DM patients complicated with CAD were compared between Sections and intra-each Section with glucose and lipid metabolism indicators.

#### Section 1

Thirty patients in Liraglutide group were daily injected with 0.6 mg of Liraglutide before breakfast. Fasting plasma glucose (FPG) was measured 7-day later. The dosage was increased to 1.2 mg/d if FPG > 7.0 mmol/L or 2-h PPG > 11.1 mmol/L, otherwise it was remained at 0.6 mg/d. After another 7 days, the dosage was increased to 1.8 mg/d if FPG > 7.0 mmol/L or 2-h PPG > 11.1 mmol/L, otherwise it remained at 1.2 mg/d. The average dose of Liraglutide was 1.2 mg/d. 30 patients in Metformin group took orally Metformin with starting dosage of 500 mg twice daily before breakfast and supper. The dosage was adjusted every other day if FPG > 7.0 mmol/L or 2-h PPG > 11.1 mmol/L. The maximum dose was 2000 mg/d, with the average dose of 1500 mg/d.

#### Section 2

Patients were was daily injected with the 0.6 mg of Liraglutide before breakfast. FPG was measured 7-day later. The dosage was increased to 1.2 mg/d if FPG > 7.0 mmol/L, or 2-h PPG >11.1 mmol/L, otherwise the patients were excluded. After another 7 days, only patients with FPG > 7.0 mmol/L or 2-h PPG > 11.1 mmol/L were remained in the study, who were randomized to either Liraglutide monotherapy (*n* = 30) or the combination therapy of Liraglutide and Metformin (*n* = 30). The dosage of Liraglutide was increased to 1.8 mg/d in the monotherapy group, while Liraglutide was maintained in 1.2 mg/d in combined therapy group. The dosage of Metformin was added to the combined therapy group according to the same strategy used in Section 1.

For patients with suboptimal glucose control at the start of the trial, concomitant use of α- glucosidase inhibitors was permitted at the discretion of the investigator.

Patients were randomly grouped according to the randomization number. The blood test, echocardiography, and blood pressure were carried out and analyzed by the technicians, who were blinded to the treatment allocation.

### Study outcomes

The primary outcomes included a total of 8 measurements of FBG (before and 2 h after 3 meals, before bedtime, and at midnight) taken in the same day once a week. Other parameters, including FBG, PPG, HbA1c, blood pressure, body mass index (BMI), TG, total cholesterol (TC), low-density lipoprotein cholesterol (LDL-C), C-reactive protein (CRP), left ventricular end-diastolic diameter (LVEDD), ejection fraction (EF) and E/A ratio were recorded at baseline before treatment and at 24-week after treatment. FBG between 5.0–7.0 mmol/L, PPG between 7.8–11.1 mmol/L and HbAlc <7.0% were considered normal.

The secondary outcomes were:1) severe hypoglycemia (requiring assistance from others); 2) moderate hypoglycemia (showing symptoms of hypoglycemia with FBG < 3.9 mmol/L, which can be treated with food intake without need of other’s assistance); 3) mild hypoglycemia (showing symptoms of hypoglycemia with FBG >3.9 mmol/L).

The adverse events, like nausea, vomiting, diarrhea, rash, red/itchy/inflamed skin, and induration or irritation of injection site skin were recorded.

### Data management and statistical analysis

Data were entered into an Excel (Micro-soft office 2000) database and were proofed for entry errors. The database was subsequently locked, imported into SPSS for Windows (IBM SPSS Statistics for Windows, Version 11.0. Armonk, NY: IBM Corp.) formatted and analyzed. BMI, FBG, TG, TC, LDL-C, PPG, HbA1c, SBP, DBP, CRP, LVEDD, EF and E/A ratio were normally distributed as continuous variables, and were analyzed with Student’s t-test between groups. Independent samples t-test was performed for the changes from baseline to week 24 of all parameters. Categorical data were analyzed by χ^2^ tests. α was set at 0.05 for all tests.

## Results

**Fig. 1:**
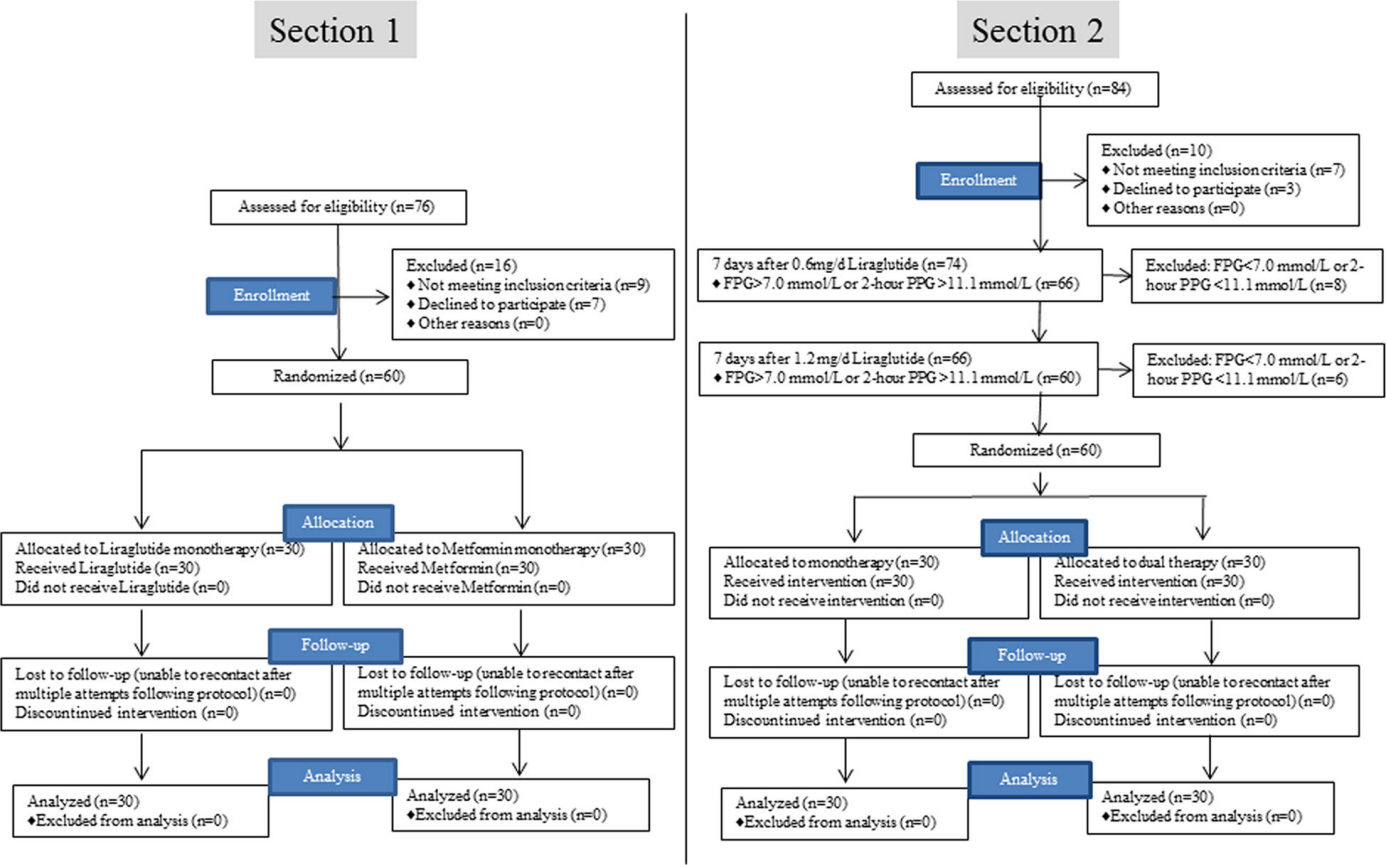
Flow chart of study design and patient selection

Once Section 1 analysis was completed, the patients for Section 2 were recruited. With 76 patients screened for Section 1, 60 met eligibility criteria and were evenly grouped into Liraglutide or Metformin monotherapy. Of the 84 patients screened for Section 2, 60 met eligibility criteria and were grouped into singled (monotherapy) or combined (dual) therapy (Fig. 1). Baseline demographic and clinical characteristics were similar between groups (Table 1). Patients in Section 1 and 2 were aged between 26 and 88 years (median, 59 years) and between 27 and 82 years (median, 60 years), respectively. The mean duration of diabetes was 8 ± 5 years in both Sections.

**Table 1:** Demographic and baseline characteristics between groups in each Section (*n* = 30 for each group)

Variables	Section 1			Section 2		
	Liraglutide monotherapy	Metformin monotherapy	*P*	Liraglutide (1.8 mg/d) monotherapy	Liraglutide plus Metformin dual therapy	*P*
Male, n (%)	16 (53.3)	17 (46.7)	0.795	16 (53.3)	17 (46.7)	0.795
Duration of diabetes (year)	8 ± 5	7 ± 4	0.396	8 ± 5	9 ± 6	0.486
Age at initial visit (year)	58 ± 15	59 ± 17	0.810	58 ± 16	57 ± 14	0.798
Pre-medication						
Statins (n)	2	3	0.640	4	4	1.000
Fibrates (n)	2	1	0.554	3	2	0.640
ARB (n)	4	3	0.688	5	5	1.000
ACEI (n)	1	2	0.554	1	0	0.313
Calcium antagonists (n)	2	2	1.000	2	3	0.640
Diuretics (n)	0	0	–	0	1	0.313
BMI (kg/m^2^)	29.56 ± 1.68	29.63 ± 1.75	0.875	29.78 ± 1.85	29.66 ± 1.78	0.799
FPG (mmol/L) HbA1c	9.3 ± 1.7	9.1 ± 2.0	0.678 0.749	9.0 ± 2.1	9.3 ± 1.7	0.546 0.758
%	9.0 ± 1.3	9.1 ± 1.1		9.3 ± 1.3	9.4 ± 1.2	
mmol/L	75 ± 14	76 ± 12		78 ± 14	79 ± 13	
PPG (mmol/L)	13.8 ± 5.1	13.7 ± 4.8	0.938	13.8 ± 4.9	13.6 ± 5.3	0.880
CRP (mg/L)	9.5 ± 2.2	9.7 ± 2.4	0.738	9.5 ± 2.2	9.7 ± 2.4	0.738
TG (mmol/L)	2.8 ± 0.7	2.9 ± 0.6	0.555	2.8 ± 0.6	2.9 ± 0.8	0.586
TC (mmol/L)	6.2 ± 0.8	6.3 ± 0.9	0.651	6.4 ± 0.8	6.3 ± 0.7	0.608
LDL-C (mmol/L)	4.1 ± 0.8	4.2 ± 0.7	0.608	4.3 ± 0.8	4.2 ± 0.7	0.608
SBP (mmHg)	145 ± 9	144 ± 8	0.651	146 ± 7	144 ± 8	0.307
DBP (mmHg)	92 ± 5	93 ± 7	0.527	94 ± 8	93 ± 7	0.608
LVEDD (mm)	55 ± 7	56 ± 9	0.633	59 ± 7	58 ± 9	0.633
EF (%)	48 ± 5	47 ± 4	0.396	46 ± 5	47 ± 4	0.396
E/A ratio	0.75 ± 0.09	0.72 ± 0.07	0.155	0.73 ± 0.06	0.75 ± 0.07	0.240

Data are mean ± SD, unless otherwise noted; *Abbreviations: BMI* body mass index, *TG* triglyceride, *TC* total cholesterol, *LDL-C* low-density lipoprotein cholesterol, *PPG* postprandial glucose, *SBP* systolic blood pressure, *DBP* diastolic blood pressure, *CRP* C-reactive protein, *LVEDD* left ventricular end-diastolic diameter, *EF* ejection fraction

### Saction1: Liraglutide vs. Metformin monotherapy

After 24 weeks, Liraglutide monotherapy significantly decreased the plasma glucose from 13.8 ± 5.1 to 7.9 ± 3.3 mmol/L, HbA1cfrom 9.0 ± 1.3% (or 75 ± 14 mmol/L) to 6.8 ± 0.8% (or 51 ± 9 mmol/L); while Metformin significantly decreased the plasma glucose from 13.7 ± 4.8 to 8.2 ± 3.5 mmol/L and HbA1c from 9.1 ± 1.1% (or 96 ± 12 mmol/L) to 6.9 ± 0.7% (or 52 ± 8 mmol/L). Obviously, no significant differences on decreases of the plasma glucose and HbA1c were seen between two treatments. Some risk factors for CAD were significantly decreased with both treatments. Plasma TG was decreased from 2.8 ± 0.7 to 2.4 ± 0.4 mmol/L, TC from 6.2 ± 0.8 to 5.4 ± 0.7 mmol/L, and LDL-C from 4.1 ± 0.8 to 3.5 ± 0.5 mmol/L in Liraglutide group; while plasma TG was significantly decreased from 2.9 ± 0.6 to 2.6 ± 0.5 mmol/L, TC from 6.3 ± 0.9 to 5.7 ± 0.8 mmol/L, LDL-C from 4.2 ± 0.7 to 3.8 ± 0.6 mmol/L in Metformin group (Tables 1 and 2). The two medications showed the equal efficacy on decreasing plasma glucose. To our surprise, Liraglutide (1.2 mg/d) alone showed better improvements on the lipid metabolism and the cardiovascular function, including TG, LDL-C, CRP, SBP, DBP, LVEDD, EF and E/A ratio (Tables 2 and 3).

**Table 2:** Clinical characteristics of patients at 24-week after medication treatment (*n* = 30 for each group)

Variables	Section 1			Section 2		
	Liraglutide monotherapy	Metformin monotherapy	*P*	Liraglutide (1.8 mg/d) monotherapy	Liraglutide plus Metformin dual therapy	*P*
BMI (kg/m^2^)	28.37 ± 1.72	28.70 ± 1.87	0.480	28.65 ± 1.77	28.42 ± 1.74	0.614
FPG c	6.0 ± 1.2	6.2 ± 1.0	0.701 0.608	6.0 ± 1.2	6.2 ± 1.0	0.486 0.633
%	6.8 ± 0.8	6.9 ± 0.7		6.8 ± 0.9	6.7 ± 0.7	
mmol/L	51 ± 9	52 ± 8		51 ± 10	50 ± 8	
PPG (mmol/L)	7.9 ± 3.3	8.2 ± 3.5	0.734	7.9 ± 3.4	7.7 ± 3.2	0.815
TG (mmol/L)	2.4 ± 0.4	2.6 ± 0.5	0.093	2.5 ± 0.4	2.4 ± 0.5	0.396
TC (mmol/L)	5.4 ± 0.7	5.7 ± 0.8	0.128	5.7 ± 0.7	5.3 ± 0.6	0.021
LDL-C (mmol/L)	3.5 ± 0.5	3.8 ± 0.6	0.040	3.7 ± 0.7	3.4 ± 0.6	0.080
CRP (mg/L)	5.1 ± 1.5	7.2 ± 1.7	<0.001	5.5 ± 1.6	5.2 ± 1.3	0.429
SBP (mmHg)	134 ± 7	141 ± 7	<0.001	135 ± 7	132 ± 8	0.127
DBP (mmHg)	86 ± 6	91 ± 6	0.002	87 ± 6	86 ± 5	0.486
LVEDD (mm)	46 ± 6	50 ± 7	0.021	52 ± 8	48 ± 7	0.044
EF (%)	53 ± 5	50 ± 6	0.040	52 ± 5	54 ± 6	0.166
E/A ratio	0.92 ± 0.08	0.77 ± 0.09	<0.001	0.88 ± 0.08	0.93 ± 0.09	0.027

Data are mean ± SD; *Abbreviations: BMI* body mass index, *TG* triglyceride, *TC* total cholesterol, *LDL-C* low-density lipoprotein cholesterol, *PPG* postprandial glucose, *SBP* systolic blood pressure, *DBP* diastolic blood pressure, *CRP* C-reactive protein, *LVEDD* left ventricular end-diastolic diameter, *EF* ejection fraction

**Table 3:** Changes of plasma HbA1c, blood lipids and cardiac function between Baseline and End of Study (week 24)

Variables	Liraglutide monotherapy	Metformin monotherapy	*P*	Liraglutide (1.8 mg/d) monotherapy	Liraglutide plus Metformin dual therapy	*P*
BMI (kg/m^2^)	−1.07 ± 0.23	−0.73 ± 0.14	<0.001	−1.13 ± 0.16	−1.20 ± 0.21	0.152
HbA1c (%)			0.278			0.093
%	−2.6 ± 0.3	−2.5 ± 0.4		−2.6 ± 0.4	−2.8 ± 0.5	
mmol/mol	−28 ± 3	−27 ± 4		−28 ± 0.4	−31 ± 5	
PPG (mmol/L)	−5.8 ± 1.7	−5.5 ± 1.6	0.484	−5.4 ± 1.5	−5.7 ± 1.6	0.459
TC (mmol/L)	−0.7 ± 0.4	−0.5 ± 0.3	0.033	−0.7 ± 0.3	−0.9 ± 0.4	0.033
LDL-C (mmol/L)	−0.6 ± 0.3	−0.4 ± 0.2	0.003	−0.5 ± 0.3	−0.8 ± 0.4	0.002
CRP (mg/L)	−4.3 ± 1.2	−2.4 ± 1.0	<0.001	−4.1 ± 0.8	−4.8 ± 1.0	0.004
SBP (mmHg)	−11 ± 4	−4 ± 2	<0.001	−11 ± 3	−12 ± 4	0.278
DBP (mmHg)	−6 ± 3	−3 ± 1	<0.001	−6 ± 3	−7 ± 4	0.278
LVEDD (mm)	−9 ± 4	−5 ± 3	<0.001	−8 ± 3	−10 ± 4	0.033
EF (%)	5 ± 3	3 ± 2	0.004	5 ± 2	7 ± 3	0.004
E/A ratio	0.16 ± 0.04	0.05 ± 0.02	<0.001	0.14 ± 0.02	0.18 ± 0.04	<0.001

*Abbreviations: BMI* body mass index, *TG* triglyceride, *TC* total cholesterol, *LDL-C* low-density lipoprotein cholesterol, *PPG* postprandial glucose, *SBP* systolic blood pressure, *DBP* diastolic blood pressure, *CRP* C-reactive protein, *LVEDD* left ventricular end-diastolic diameter, *EF* ejection fraction

### Section 2: Monotherapy vs. combined therapy

Due to a better efficacy of Liraglutide shown in Section 1, higher dose of Liraglutide (1.8 mg/d) alone was chosen for study in Section 2, and Liraglutide (1.2 mg/d) plus Metformin was selected for combined therapy. After 24-week treatment, plasma glucose of 7.9 ± 3.4 mmol/L in monotherapy and 7.7 ± 3.2 mmol/L in dual therapy, and HbA1c of 6.8 ± 0.9% (or 51 ± 9 mmol/L) in monotherapy and 6.7 ± 0.7% (or 50 ± 8 mmol/L) in dual therapy were significantly decreased when compared with the levels of baseline, in which averages of plasma glucose and HbA1c from two sections at baseline vs. 24-week treatment were 9.2 ± 0.2 vs. 6.8 ± 0.1%. However, there was no significant difference on glycemic control between groups in Section 2 (Tables 2 and 3).

However, in section 2, combined treatment of Liraglutide and Metformin showed the better improvements only on TC, LVEDD and E/A ratio when compared with Liraglutide (1.8 mg/d) alone treatment (Table 2).

In addition, regarding the adverse events associated with each treatment, less events such as transient anorexia, nausea, and vomiting were observed in the dual therapy than in Liraglutide monotherapy, which were shown in Table 4.

**Table 4:** Adverse Events Caused with Treatments (n (%))

Adverse Events	Section 1			Section 2		
	Liraglutide monotherapy *n* = 30	Metformin monotherapy *n* = 30	*P*	Liraglutide (1.8 mg/d) monotherapy *n* = 30	Liraglutide plus Metformin dual therapy *n* = 30	*P*
Transient anorexia, nausea or vomit	5 (16.7)	7 (23.3)	0.519	6 (20.0)	14 (46.7)	0.029
Transient diarrhea	2 (6.7)	2 (6.7)	1.000	3 (10.0)	1 (3.3)	0.612
Hypoglycemia	1 (3.3)	1 (3.3)	1.000	4 (13.3)	3 (10.0)	1.000
Injection site skin induration and irritation	1 (3.3)	0 (3.3)	0.313	1 (3.3)	2 (6.7)	1.000

## Discussion

Both Liraglutide and Metformin have been extensively studied on the basis of glycemic control. The current study was focused on the cardiovascular function analyses with Liraglutide and/or Metformin treatment in T2DM patients complicated with CAD. We have found that, with comparable glycemic levels, both Liraglutide monotherapy and Metformin monotherapy could significantly improve plasma lipid profile, BMI, CRP, and cardiac functions. However, Liraglutide (1.2 mg/d) monotherapy significantly improved the LDL-C, CRP, BP, LVEDD, EF percentage and E/A ratio, compared with Metformin monotherapy. Based on the results, we further compared the higher dose of Liraglutide (1.8 mg/d) monotherapy with the dual therapy of low dose Liraglutide (1.2 mg/d) plus Metformin. To our surprise, not only the dual therapy but also the higher dose of Liraglutide (1.8 mg/d) monotherapy showed less improvements on the lipid metabolism and the cardiovascular function, compared with Liraglutide (1.2 mg/d) monotherapy. It is understandable that as analogue of GLP-1, Liraglutide may slow the rate of absorption of nutrients into the blood stream by reducing gastric emptying and may directly reduce food intake, including decreasing lipids intake and absorption. A recent study of 36-month LEADER trial revealed that Liraglutide improved cardiovascular outcomes in T2DM patients [12]. It was also reported that an addition of 5-week infusion of GLP-1 to standard therapy in patients with New York Heart Association class III/IV heart failure significantly improved cardiac function, as indicated by left ventricular ejection fraction, maximum myocardial ventilation oxygen consumption, and 6-min walk distance [11]. Pre-clinical and clinical studies have also revealed the beneficial effect of Liraglutide on lipid metabolism/profile and endothelial function in T2DM patients [12–14]. Possible underlying mechanisms include alleviated oxidative stress and cardiomyocytes apoptosis by GLP-1, prevention of micro-vascular diseases related to diabetes, protection of cardiac micro-vascular endothelial cells against hypoxia/re-oxygenation injury, regulation of calcium homeostasis and electrophysiological activities of cardiomyocytes [12, 13, 15–19].

GLP-1’s actions are mediated through the G-protein-coupled GLP-1 receptor. GLP-1 or GLP-1 receptor agonist decreases food intake, slows gastric emptying, and reduces body weight through its effects on energy expenditure. As well reviewed, liraglutide has been demonstrated to reduce food intake, promote weight loss, and improve indices of metabolic function in both animal and human studies, and the primary mechanisms associated with these effects are proposed to be due to actions of GLP-1 on peripheral and central pathways that affect food intake and metabolism via hindbrain and hypothalamic activation, as well as those brain areas associated with motivation and reward processes [20]. In patients with Type 2 diabetes, well controlled with metformin monotherapy, addition of liraglutide improves several cardiovascular risk markers beyond glycaemic control [21]. These studies, together with our data, support the effect of Liraglutide on improving the cardiovascular function of T2DM patients besides glycemic control.

The ability of Metformin to reduce hepatic glucose production and increase skeletal muscle glucose uptake has made it as one of the major first-line therapies for T2DM [22, 23]. It was also reported that Metformin could reduce insulin resistance, lower blood pressure, improve circulating lipid profile, and prevent myocardial infarction and stroke in patients with diabetes [24–31]. In line with these results, the current study also demonstrated that Metformin improve lipid profile and cardiovascular function beyond glycemic control. It was reported that Metformin could inhibit the production of triglycerides and cholesterol, and stimulates fatty acid oxidation, preventing the progression of nonalcoholic fatty liver disease [32, 33]. A recent report demonstrated a significant association between metformin response and TG, TC, HDL-C and LDL-C [34]. The glucose-lowering property alone cannot account for cardiovascular benefit [26]. Accumulating evidences suggest the inhibition of pro-inflammatory responses, reduction of smooth muscle cell contractility, or increase of nitric oxide as possible mechanisms [35, 36].

Many studies were conducted to investigate the synergistic effect of different drugs. Liraglutide or Metformin has previously been studied to combine with other drugs such as thiazolidinedione (TZD) and sulphonylurea [37–39]. All these dual and triple therapy showed, not only the more effective on decreasing blood glucose level, but also on reducing body weight, BP, BMI, compared with monotherapy. Theoretically, combined dual or triple drug therapy should produce a synergetic effect, which must be better than monotherapy. However, it remains unknown about the mechanism of the Liraglutide (1.2 mg/d) showed the better improvements on the lipid profiles and the cardiac function than either Liraglutide (1.8 mg/d) or dual therapy, so future studies are needed.

The most common adverse events associated with GLP-1 RAs were nausea, vomiting, and diarrhea, the causes of which were thought to be gastric emptying or the involved central nervous system. [40] To achieve similar glycemic control, a lower dose of Liraglutide was needed in the dual therapy. Not surprisingly, with similar glycemic control in T2DM patients complicated with CAD, the occurrence of adverse events were significantly lower in the dual therapy, suggesting underlying advantage of dual therapy over monotherapy.

The limitation of the study were: 1) the sample size was relatively small, indicating less statistical power, lower chance of discovering genuine effects, and the possible exaggerated estimate [22]; 2) only one time point (24 weeks after treatment) was assessed, while T2DM complicated with CAD is a chronic disease, a longer-term observation would be of good value.

3) Recently, GLP-1 based therapies also have the potential to be linked to cancer, specifically thyroid cancer and pancreatic cancer [41]. However, no cancer risk was found with Liraglutide treatment in the present study.

## Conclusion

In summary, the Liraglutide (1.2 mg/d) monotherapy showed the better effects than either Metformin alone, or combination of Liraglutide and Metformin on lipid metabolism and cardiovascular function in patients with similar glycemic control.

## Abbreviations

BMI: Body mass index
BP: Blood pressure
CAD: Coronary artery disease
CRP: C reactive protein
EF: Ejection fraction
FBG: Fasting blood glucose
FFA: Free fatty acid
GLP-1: Glucagon-like peptide-1
HbA1c: Glycated hemoglobin
LDL-C: Low-density lipoprotein cholesterol
LVEDD: Left ventricular end-diastolic diameter
PPG: Postprandial glucose
RAs: Receptor agonists
RLP-C: remnant lipoprotein cholesterol
T2DM: Type 2 diabetes mellitus
TC: Total cholesterol
TG: Triglyceride
TZD: Thiazolidinedione
UCG: Ultrasound cardiography
